# P01.43. The calm mouse: an animal model of stress reduction

**DOI:** 10.1186/1472-6882-12-S1-P43

**Published:** 2012-06-12

**Authors:** B Gurfein, A Stamm, P Bacchetti, M Dallman, N Nadkarni, J Milush, C Touma, R Palme, C Pozzo Di Borgo, G Fromentin, R Lown-Hecht, J Konsman, M Acree, M Premenko-Lanier, N Darcel, F Hecht, D Nixon

**Affiliations:** 1 University of California, San Francisco, San Francisco, USA; 2 AgroParisTech, Paris, France; 3 Max Planck Institute of Psychiatry, Munich, Germany; 4 University of Veterinary Medicine, Vienna, Austria; 5 Victor Segalen Bordeaux 2 University, Bordeaux, France

## Purpose

Chronic stress is associated with negative health outcomes and is linked with neuroendocrine changes, suppressed immunity, and central nervous system neuropathology. While human studies have illustrated the benefits of stress reduction, mechanistic understanding of how decreasing stress affects health and disease progression remains unclear. Furthermore, prior animal studies have focused primarily on increasing stress, and few animal models of stress reduction have been fully developed.

## Methods

We have developed a “calm mouse model” with caging enhancements designed to reduce murine stress. Male BALB/c mice were divided into four groups (n=10/group): Control (Cntl), standard caging; Calm, large caging to reduce animal density, a cardboard nest box for shelter, paper nesting material to promote innate nesting behavior, and a polycarbonate tube to mimic tunneling; Control Exercise (Cntl Ex), standard caging with a running wheel, known to reduce stress; and Calm Exercise (Calm Ex), Calm caging with a running wheel.

## Results

Calm, Cntl Ex, and Calm Ex animals exhibited significantly less corticosterone production than Cntl (Day 49: Calm, M> 20.5 ng corticosterone metabolites/0.05g feces (CM), CI95 11.7 to 29.4, p< 0.0001; Cntl Ex, M> 22.5ng CM, CI95 13.4 to 31.5, p<0.0001; Calm Ex, M> 21.8 CM, CI95 11.7 to 32.0, p=0.0003). Calm animals gained greater body mass than Cntl, although they had similar weekly energy intake. We also observed changes in body composition, behavior, spleen mass, and immune function. Lastly, our *in vitro* studies showed that Calm Ex animals had innate and adaptive immune responses that were more sensitive to acute stress.

## Conclusion

Our data indicate that both Calm and exercise caging generated reductions in physiologic stress measures in mice. Collectively, the Calm model represents a promising approach to studying the biological effects of stress reduction in the context of health and in conjunction with existing disease models.

